# Musculoskeletal pain and co-morbid insomnia in adults; a population study of the prevalence and impact on restricted social participation

**DOI:** 10.1186/s12875-017-0593-5

**Published:** 2017-02-07

**Authors:** Shula Baker, John McBeth, Carolyn A. Chew-Graham, Ross Wilkie

**Affiliations:** 1 Research Institute for Primary Care & Health Sciences, Keele University, ST5 5BG Keele, UK; 2 Arthritis Research UK Centre for Epidemiology, University of Manchester, Manchester, M13 9PT UK; 3 Collaboration for Leadership in Applied Health Research and Care, West Midlands Birmingham, UK

**Keywords:** Musculoskeletal pain, Insomnia, Social activity, Comorbidity

## Abstract

**Background:**

Comorbidity is common in patients consulting in primary care. Musculoskeletal pain and insomnia each increase the risk of the other. Co-occurrence may pose an increased burden on well-being. However, the prevalence and impact of co-existing pain and insomnia in adults living in the community who may present to primary care is unclear. The aim of this study was to report the prevalence of pain and insomnia in adults registered with primary care, and to examine the impact of co-occurrence on social activities.

**Methods:**

This population-based prospective cohort study of adults aged ≥18 years (n = 1181) used health survey data collected via baseline and 12 month follow-up questionnaires. Baseline data on pain, insomnia (4 symptoms: delayed sleep onset, difficulty maintaining sleep, early waking and non-restorative sleep) and putative confounders and social activity restriction at follow up was collected. Associations between baseline pain, insomnia and restricted social activities (RSA) at 12 months were examined using logistic regression, with adjustment for confounders. Interaction terms between pain and each insomnia symptom were examined in final models.

**Results:**

Mean respondent age was 49.6 (SD ± 15.2) years, 55.7% were female. At baseline, 880 (74.5%) reported pain, 122 (10.3%) delayed sleep onset, 298 (25.2%) difficulty maintaining sleep, 188 (15.9%) early wakening, and 215 (18.2%) reported non-restorative sleep. At follow-up 200 (16.9%) reported RSA. Pain and each insomnia symptom were associated with RSA at 12 month follow-up; pain [unadjusted odds ratio (OR:2.3;95%CI:1.5,3.5), delayed sleep onset (OR:6.1;95%CI:4.0,9.1), difficulty maintaining sleep (OR:3.2;95%CI:2.3,4.4), early wakening (OR:4.1;95%CI:2.9,5.9), and non-restorative sleep (OR:4.0; 95%CI:2.8,5.8). Only delayed sleep onset (OR:2.6;95%C:1.5,4.5) remained significantly associated with restricted social activities in the fully adjusted model. There was a significant interaction between pain and delayed sleep onset (OR:0.3;95%CI:0.1,0.99; p = .049) and restricted social activity at 12 months in the final multivariable model.

**Conclusions:**

Pain and insomnia commonly co-occur, resulting in greater impact upon subsequent functional ability. Delayed sleep onset is the insomnia symptom most strongly associated with reduced functional ability. Clinicians should be aware of the common co-occurrence of insomnia symptoms, inquire about sleep in patients consulting with pain, and offer interventions that target both sleep and pain.

## Background

Musculoskeletal pain is common in adults and is a frequent reason for consultation to primary health care [1]. One quarter to one third of the general population report low back, hip or shoulder pain and one in five experience chronic pain (i.e. pain that lasts for three months or more) [2, 3]. Musculoskeletal pain impacts on physical and mental health, and mortality risk [4, 5]. Thirty percent of adults in the general population report significant sleep disturbance and 6% to 10% meet diagnostic criteria for insomnia [6], defined as difficulty initiating or maintaining sleep, or nonrestorative sleep, for at least 1 month [7]. Left untreated, insomnia is associated with an increased incidence of depression, anxiety [8] and worse physical health [9]. Insomnia frequently occurs in patients with chronic pain with the prevalence of co-occurrence ranges between 50% and 88% [10, 11]. However, the prevalence and impact of co-existing musculoskeletal pain and insomnia in adults living in the community who may present to primary care is unclear.

Musculoskeletal pain and insomnia have a reciprocal relationship, with each condition increasing the risk of the other which may augment the burden on health and well-being [12]. The mechanisms underlying the association appear to be complex and multi-factoral. Sleep disruption may be attributed to musculoskeletal pain arising from painful stimuli during sleep, which can induce microarousal and increase wakefulness [11]. There is also evidence to suggest that recurrent sleep deprivation and disruption (especially disruption of slow wave sleep) for three consecutive nights or more can decrease an individual’s pain threshold, amplify negative mood and produce somatic symptoms [10, 11].

Social participation involves taking part in social activities and hobbies as well as fulfilling social roles such as being a worker, carer or community member [13]. Maintaining social participation is a clinically important outcome, potentially modifiable by intervention, and a target for intervention in primary care [14]. Restricted social activity is associated with higher rates of morbidity and mortality and lower life satisfaction and health-related quality of life [15, 16]. The aim of this prospective cohort study was to report the prevalence of co-morbid pain and insomnia in community-dwelling adults registered with primary care and its association with restricted social activities.

## Methods

### Study design and procedure

The study was a population-based prospective cohort study exploring headache prevalence in the general adult population. Five general practices were selected from 15 practices constituting the North Staffordshire GP Research Network, to contain a mix of urban and rural settings, and a spread of social class. The practice age/sex registers were downloaded with unique identifiers and sampling was conducted by obtaining randomly generated samples of 1000 persons, aged 18 and over, from each of 5 general practices in North Staffordshire (total sample of 5000). The sample size was determined based upon expected headache prevalence. As this study was concerned with pain in any body part, so included many additional pain sites, the sample size was also sufficient for the purpose of this research question. After excluding those individuals who had recently moved, died or were unable to participate due to ill health (N = 243,4.9%) a total of 4757 persons were invited to take part in the study. In the UK over 95% of the population are registered with a general practice and provide representative samples of the general population [17]. Ethical approval was obtained from the North Staffordshire Local Research Ethics Committee. All participants provided informed consent to participate in the study.

### Baseline survey

Potential participants were mailed a baseline questionnaire that collected data on pain, insomnia and putative confounders.

#### Assessment of musculoskeletal pain

To assess musculoskeletal pain participants were asked to indicate the site of any pain lasting one day or longer that they had experienced during the last month on a blank body manikin (front and back views). Pain manikins are valid and reliable tools for recording pain prevalence in self-administered questionnaires [18, 19]. Using their reports of pain, participants were classified into ‘any pain’ (any shading of pain on the manikin) or ‘no pain’ (no shading) groups.

#### Identification of insomnia

The four-item Estimation of Sleep Problems Scale [20] was used to examine sleep quality and identify insomnia. The scale asks about recent problems with sleep and contains items on the most commonly occurring symptoms of poor sleep quality: delayed sleep onset (‘During the past four weeks did you have trouble falling asleep?’); sleep maintenance (‘During the past four weeks did you wake up several times per night?’); early wakening (‘During the past four weeks did you have trouble staying asleep, including waking up far too early?’); and non-restorative sleep (‘During the past four weeks did you wake up after your usual amount of sleep feeling tired and worn out?’). Participants indicate the frequency in the past month that they have experienced difficulties in each of the four sleep components on a 3-point scale ranging from 0 to 2 (0 = not at all; 1 = on some nights; 2 = on most nights). For this analysis “on most nights” was used to define the presence of each respective sleep problem. This method of determining those with insomnia has been validated for use both in individuals with pain [21] and the general population [22].

#### Putative confounders

Putative confounders were demographics (age, gender), and socio-economic status (occupational class: manual (skilled manual, partly-skilled or unskilled roles professional/managerial, semi-routine, routine) / non-manual (professional, managerial or skilled non-manual roles)), anxiety, depression and physical health related quality of life. Levels of anxiety and depression were measured using the Hospital Anxiety and Depression Scale (HADS) [23]. The HADS performs well in assessing severity and identifies cases of anxiety and depression in hospital practice, for which it was first designed, primary care and in the general population [24]. The sensitivity and specificity of each scale to detect cases when clinical diagnosis is considered as the gold standard ranges between 70 and 80% [24, 25]. The HADS, along with the Geriatric Depression Scale, have been identified as the best instruments to measure mood and behaviour in older adults in observational studies and trials, scoring highly for practicality, feasibility, psychometric properties and relevant content [26]. The HADS scale consists of 14 items scored on a Likert scale of 0–3: 7 items ask about symptoms of anxiety and give a total score of 0–21, and 7 items ask about symptoms of depression, giving a total score of 0–21. Higher scores represent more frequent symptoms of depression/anxiety. For both scales scores of 0–7 were classified as a non-case, 8–21 a probable case using guidelines by the original authors [23]. Scores of ≤7 are considered ‘normal’ in the general adult population, while those of 8 or more are suggestive of a disorder [24]. HADS has been shown to have good sensitivity and specificity when a cut-off of ≤7 is used to identify probably cases of depression and anxiety in samples of primary care patients [24].

Physical health related quality of life was measured using the Medical Outcomes Short-Form 12 (SF-12) physical component summary score [27]. Computation of the PCS component was achieved by multiplying each indicator variable by a respective physical regression weights provided as part of the SF-12 scoring algorithm [27]. As in other studies [28], PCS score tertiles were then used in the analyses for the ease of interpretation, with the highest third used as the referent group.

#### Identifying restricted social activity at follow-up

Participants who returned a baseline questionnaire, and who agreed to further contact, were mailed a follow-up questionnaire 12 months later. A single item from the SF-12 was used to measure RSA at 12 month review; ‘Has your health limited your social activities (like visiting friends or close relatives)?’ RSA was defined as responses of ‘All’/’Most’/’A good bit’/’Some of the time’, and those responding ‘A little bit of the time’ or ‘None of the time’ were defined as not having RSA. This point was chosen based upon the extent of restriction reported being judged as likely to reduce functional ability, and the total number reporting RSA exceeding 10% of the overall sample. Baseline RSA was included as a putative confounder, and was measured and categorised using the same method.

#### Statistical analysis

A complete-case analysis was conducted that included only those participants with complete data at baseline and follow-up. First, the distribution of baseline variables were examined by pain and insomnia status with differences tested for significance using Chi-square or Kruskal Wallis tests where appropriate. Bootstrapped (*n* = 1000) 95% confidence intervals were calculated for the prevalence of pain and each insomnia symptom to provide an estimation for the study population. Univariate logistic regression models examined the relationship between pain and insomnia, and RSA at 12 month follow-up, adjusting for age, gender, and occupational class. Pain and insomnia were then included in the same multivariate model which was cumulatively adjusted for: i) putative confounders age, gender and occupational class (model 1), ii) anxiety, depression and physical health related quality of life (model 2), iii) baseline social participation (model 3). Finally, to determine whether the association between pain and RSA at 12 month follow-up was moderated by insomnia, an interaction term between pain and each insomnia symptom was included separately adjusting for all confounders (model 4), and then all interaction terms were included in the same model. Outliers were tested for by looking at for studentized residuals larger than 3 for all variables in the model. None were found.

Continuous covariates were standardised prior to entry into the regression analysis. Results are reported as odds ratios (OR) with 95% confidence intervals (95%CI). Model goodness of fit was examined for each model using the area under the Receiver Operating Characteristic curve (AUROC) to examine predictive power, and the Hosmer-Lemeshow test to highlight the goodness-of-fit to the data. AUROC values are measures of a model’s ability to discriminate between those with RSA at 12 months and those without. Traditionally values of 0.7 or more represent moderate accuracy/discrimination [29]. Analysis was performed using SPSS 19.0 and Stata 13.0 for Windows

## Results

**Fig. 1:**
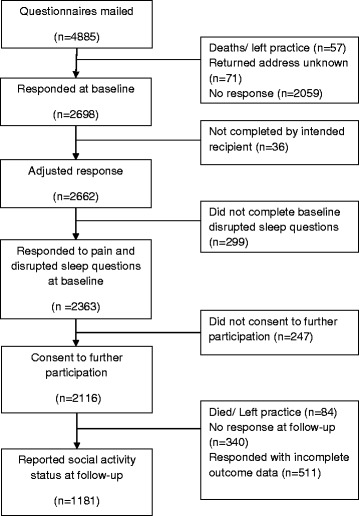
Flow diagram of participants

Of 4757 participants who were eligible to take part, 2662 (56.0%) returned a completed questionnaire at baseline. Compared to participants, non-participants were younger (mean age: 47 years cf. 51 years; p < 0.001) and more likely to be male (52 cf. 42%; *p* < 0.001). After excluding those who refused further contact (*n* = 247), decedents (*n* = 84), those who did not respond at follow-up (*n* = 340), or had incomplete data (*n* = 810), 1181 persons were available for analysis at follow up (Fig. 1). Comparison to UK Census data [30] showed the analytical sample to be similar in terms of gender (55.7 female cf. 51.9%) with fewer young adults (19.1% aged 18–34 cf. 29.4%) and more middle aged adults (61.4% aged 35–64 cf. 50.0%).

### Participant characteristics

Participant mean age was 49.6 (Standard Deviation (SD) ±15.2) years and 658 (55.7%) were female (Table 1). At baseline, 880 (74.5%) reported pain and 411 (34.8%) reported one or more insomnia symptoms; 122 (10.3%) reported delayed sleep onset, 298 (25.2%) difficulty maintaining sleep, 188 (15.9%) early wakening, and 215 (18.2%) non-restorative sleep. The proportion of individuals with each respective sleep problem who reported additional sleep problems is shown in Table 2. 349 (29.6%) responders reported both pain and insomnia. This was 39.7% of those who reported pain, and 84.9% of those who reported insomnia. At 12 month follow-up, 200 (16.9%) reported RSA. Of this group, 109 (54.5%) reported pain and insomnia at baseline, 62 (31.0%) reported pain only, 17 (8.5%) reported one or more insomnia symptoms and 12 (6.0%) reported neither pain nor insomnia.

**Table 1:** Participant characteristics by baseline pain and insomnia status

	Overall	No pain or insomnia	Pain only	Insomnia only	Pain and insomnia	*p*-value
Group size						*p* < 0.001
(%)	1181 (100%)	239 (20.2%)	531 (45.0%)	62 (5.2%)	349 (30.0%)	
Age^K^						*p* = 0.015
Median (IQR)	49.6 (± 15.2)	49.4 (±15.3)	48.3 (±14.8)	52. 1 (±15.0)	51.4 (±15.8)	
Gender^C^						*p* = 0.003
Female No. (%)	658 (55.7%)	122 (51.0%)	277 (52.2%)	41 (66.1%)	218 (62.5%)	
Occupation^C^ Manual (%)						*p* = 0.903
Delayed sleep onset^C^ Restricted No. (%)	122 (10.3%)	-	-	18 (29.0%)	104 (29.8%)	
Difficulty maintaining sleep^C^ Restricted No. (%)	298 (25.2%)	-	-	48 (77.4)	250 (71.6%)	*p* = 0.347
Early waking^C^ Restricted No. (%)	188 (15.9%)	-	-	33 (53.2%)	155 (44.4%)	*p* = 0.199
Non-restorative sleep^C^ Restricted No. (%)	215 (18.2%)	-	-	26 (41.9%)	189 (54.2%)	*p* = 0.076
Baseline RSA^C^ Restricted No. (%)	203 (17.2%)	11 (4.6%)	53 (10.0%)	10 (16.1%)	129 (37.0%)	*p* < 0.001
Baseline SF-12 physical function^A^ Median PCS score. (IQR)	48.5 (± 10.5)	54.3 (±4.7)	49.4 (±8.9)	51.2 (±10.0)	42.5 (±12.6)	*p* < 0.001
Baseline Anxiety^C^ Possible/probable No. (%)	460 (39.0%)	38 (15.9%)	159 (29.9%)	27 (43.5%)	236 (67.6%)	*p* < 0.001
Baseline Depression^C^ Possible/probable No. (%)	206 (17.4%)	38 (5.4%)	45 (8.5%)	15 (24.2%)	133 (38.1%)	*p* < 0.001
12 month review RSA^C^ Restricted No. (%)	200 (16.9%)	12 (5.0%)	62 (11.7%)	17 (27.4%)	109 (31.2%)	*p* < 0.001

*RSA* restricted social activity

^A^ One-way ANOVA analysis, ^K^- Kruskal Wallis analysis, ^C^- Chi square analysis

**Table 2:** The number and proportion of individuals with each insomnia symptom and pain (by column) who reported each of the other insomnia symptoms (by row)

Symptom, number reporting (%)	Pain *N* = 880 74.5%	Delayed sleep onset *N* = 122 10.3%	Poor sleep maintenance *N* = 298 25.2%	Early wakening *N* = 188 15.9%	Non-restorative sleep *N* = 215 18.2%
95% CI for population prevalence	71.9-77.1%	8.4%-2.1%	22.7-28.1%	13.8-17.9%	15.9-20.5%
Pain	-	104 (85.2%)	250 (83.9%)	155 (82.4%)	189 (87.9%)
Delayed sleep onset	104 (11.8%)	-	94 (31.5%)	89 (47.3%)	74 (34.4%)
Poor sleep maintenance	250 (28.4%)	94 (77.0%)	-	175 (93.1%)	127 (59.1%)
Early wakening	155 (17.6%)	89 (73.0%)	175 (58.7%)	-	102 (47.4%)
Non-restorative sleep	189 (21.5%)	74 (60.7%)	127 (42.6%)	102 (54.3%)	-
No other sleep problems	62 (31.0%)	15 (12.3%)	91 (30.5%)	7 (3.7%)	76 (35.3%)

### The association between baseline pain, insomnia symptoms and restricted social activity

Pain and all four insomnia symptoms were associated with RSA at 12 month follow-up; baseline pain [unadjusted odds ratio (OR:2.3;95%CI:1.5,3.5), delayed sleep onset (OR:6.1;95%CI:4.0,9.1), difficulty maintaining sleep (OR:3.2;95%CI:2.3,4.4), early wakening (OR:4.1;95%CI:2.9,5.9), and non-restorative sleep (OR:4.0;95%CI:2.8,5.8). All symptoms were statistically significant (*p* < .001).

When pain and insomnia symptoms were included in the multivariable model with adjustment for age, gender and occupational class (model 1), the associations attenuated; difficulty maintaining sleep (OR:1.3;95%CI:0.8,2.1;*p* = .173) and early wakening (OR:1.5;95%CI:0.8,2.6;*p* = .265) were not significantly associated with RSA at 12 month follow-up. When adjusted for comorbidity (model 2), the associations between RSA at 12 month follow-up and pain and non-restorative sleep attenuated to non-significance (*p* = .918). Delayed sleep onset (OR:2.6;95%CI:1.5,4.5;*p* = .001) remained significantly associated with RSA in the fully adjusted model (model 3). Results for multivariable analyses are given in Table 3.

**Table 3:** Association between baseline pain and insomnia symptoms and restricted social activity at follow-up

	Model I	Model II	Model III	Model IV
Baseline Pain	1.8* (1.2,2.9)	1.1 (0.7,1.8)	1.1 (0.6,1.8)	1.3 (0.7,2.4)
Delayed sleep onset	2.9** (1.8,4.8)	2.4** (1.4,4.1)	2.6** (1.5,4.5)	7.6** (2.3,25.4)
Difficulty maintaining sleep	1.3 (0.8,2.1)	1.1 (0.7,1.8)	1.0 (0.6,1.6)	1.0 (0.6,1.6)
Early waking	1.5 (0.8,2.6)	1.4 (0.8,2.5)	1.4 (0.7,2.5)	1.3 (0.7,2.5)
Non-restorative sleep	2.0** (1.3,3.1)	1.0 (0.6,1.6)	0.9 (0.6,1.5)	0.9 (0.6,1.5)
Age	1.5** (1.2,1.8)	1.2 (0.98,1.5)	1.2 (0.9,1.5)	1.2 (0.9,1.5)
Gender	1.2 (0.97,1.4)	1.1 (0.9,1.4)	1.2 (0.97,1.4)	1.2 (0.96,1.4)
Manual occupation	1.2 (0.98,1.4)	1.1 (0.9,1.3)	1.0 (0.8,1.2)	1.0 (0.8,1.2)
Possible/probable Depression	-	1.8** (1.5,2.1)	1.5** (1.3,1.8)	1.5** (1.2,1.8)
Possible/probable Anxiety	-	1.3* (1.0,1.6)	1.2 (0.9,1.4)	1.2 (0.9,1.5)
Poor physical function (Lowest tertile^a^)	-	2.7** (1.7,4.4)	1.9* (1.1,3.2)	2.0** (1.2,3.3)
Moderate physical function (mid tertile^a^)	-	1.3 (0.8,2.2)	1.2 (0.7,2.1)	1.2 (0.7,2.0)
Baseline RSA	-	-	4.6** (3.0,7.1)	4.6** (3.0,7.1)
Pain x delayed sleep onset interaction	-	-		0.3* (0.1,0.99)
Model fit: AUROC	0.730	0.805	0.829	0.830
Hosmer-Lemeshow test	*p* = .308 (83.2%)	*p* = .435 (85.4%)	*p* = .394 (86.5%)	*p* = .414 (86.6%)

Multivariate model I: Pain, each insomnia symptom, and sociodemographic factors. Model II: Pain, each insomnia symptom, sociodemographic factors, anxiety, depression and physical health related quality of life. Model III: Pain, each insomnia symptom, sociodemographic factors, anxiety, depression, physical health related quality of life and baseline social participation. Model IV: Model 3 plus the inclusion of an interaction term between pain and delayed sleep onset. All values are Exp (beta) with 95% confidence intervals. * *p* < 0.05, ** *p* < 0.01, − variable not included in the model

^a^Referent group was those participants in the tertile with the highest physical function scores

AUROC – Area Under the Receiver Operator Characteristic curve. Hosmer & Lemeshow test reported as p-value (insignificant p-value indicates good fit) and proportion of cases correctly classified

Interactions between pain and difficulty maintaining sleep, early wakening and non-restorative sleep were none significant predictors of RSA at 12 month follow-up (*p* > 0.05). Although significant, the coefficient for the interaction term between pain and delayed sleep onset in the final multivariable model was less than 1 (OR:0.3;95%CI:0.1,0.99;*p* = .049), indicating the combined effect to be less than that expected were the effect of the two factors to be totally independent of each other. When all interaction terms were included in the same model, the odds ratio for pain became insignificant (OR:1.6;95%CI:0.8,3.0;*p* = .180), and only delayed sleep onset remained significant of the insomnia symptoms (OR:2.6;95%CI:1.5,4.5;*p* = .001). In addition to delayed sleep onset, baseline depression (OR:1.5;95%CI:1.3,1.8;*p* < .001), low physical health related quality of life (OR:1.9;95%CI:1.2,3.2;*p* = 0.01), and baseline RSA (OR:4.6;95%CI:3.0,7.1;*p* < .001) were independently associated with RSA at follow-up in the final multivariable model.

## Discussion

The study reports the extent of co-occurrence of pain and insomnia in a primary care population, and the extent of its association with problems getting out and about, and engaging in social activities. The findings highlight the common occurrence of musculoskeletal pain and insomnia in adults in primary care patients; almost one third patients reported co-existing pain and insomnia. More than four in every five respondents with insomnia reported pain. Baseline pain and each sleep problem were associated with RSA at 12 month follow-up. The association between RSA at follow-up and pain, difficulty maintaining sleep, early wakening and non-restorative sleep attenuated with adjustment for depression, anxiety, physical health related quality of life and baseline RSA. However, delayed sleep onset was significantly associated with RSA at 12 month follow-up following adjustment for all confounders.

There was no multiplicative interaction between pain and insomnia defined by difficulty maintaining sleep, early wakening or non-restorative sleep. However there was evidence that the relationship between baseline pain and RSA at follow up was augmented by the presence of an insomnia symptom. For example, having pain but not insomnia was not significantly associated with RSA at follow-up (*p* = .180), whereas this increased to a greater than 7-fold increase in odds of RSA when delayed sleep onset was present (OR:7.4;95%CI:4.2,13.0).

The prevalence of insomnia symptoms in this study is comparable with the 10-40% reported in other population surveys [31, 32]. The prevalence of pain (72%) was high but is comparable to that reported in previous studies of community-dwelling adults [33–35]. The levels of co-occurring insomnia and pain were comparable with other studies [36, 37]. The association between musculoskeletal pain and RSA and insomnia and RSA have been reported in older adults but not in the general adult population. A systematic review of available literature (details available from the corresponding author) found no comparable population studies that have reported on the impact of estimates of co-occurring pain and insomnia and their impact on restricted social activity.

This study includes a number of strengths and limitations. The 1181 participants were recruited from general practice registers, which provide a sampling frame representative of the general population [17]. Furthermore, the response rates were comparable with other population based prospective studies that have used postal questionnaires. As in all longitudinal studies there was some attrition and missing data. Responders to the baseline survey were more likely to be younger and female. Those included in the analysis, compared to those who responded at baseline but not to the follow-up questionnaire, were less likely to have pain at baseline (p = 0.002) and more likely to be younger (p < 0.001), but they were no more likely to have insomnia at baseline (p = 0.67) or be female (p = 0.89). Non-participation bias may occur but it is unknown if the relationship between pain, insomnia and RSA in those who did not respond is different to those included in the analysis. The generalizability of the data may be limited by the characteristics of the study sample; the area covered by the study is more deprived in terms of health, education, and employment, but has fewer barriers to housing and services, than England as a whole.

The available data covered potential confounders of the relationship between pain and insomnia and RSA, however other potential confounders of the relationship have not been included (e.g. educational attainment). The questionnaires used to measure insomnia, anxiety, depression and RSA have been validated for use in general population samples and in postal surveys. RSA was operationalised as a binary measure, produced by dichotomising the six-level of responses to a single question. A sensitivity analysis, performed to check whether the choice of cut-off affected the relationship between sleep and RSA, found the association was not dependent on the cut-off used to define RSA. Pain was assessed using blank body manikins, a standard data-capture method used in postal surveys. This method has been shown to be a valid and reliable assessment of pain in mid-life adults, although the validity of manikin-derived pain in older people is less clear. High levels of inter-rater reliability for pain scoring (Kappa >0.60) has been demonstrated using this data-capture method [20].

In the pilot work for this study, there were some responders who reported pain interfering with work and but did not indicate pain on the manikin. This may have occurred because pain that interferes with work may not last for one day or longer and would not be reported on the manikin. The high prevalence of pain suggests that recall bias may be present, however the estimates are comparable with other studies that have measured the same phenotype (any pain that have lasted for a day or longer in the last month). It is also possible that pain captured includes that which is not musculoskeletal in origin which would inflate the prevalence estimates, however the majority of pain lasting one day or longer that is reported by adults in primary care is likely to be musculoskeletal in origin [35]. It is also possible that pain intensity may influence the association of musculoskeletal pain upon subsequent social activity restriction, as musculoskeletal pain intensity has been shown to predict greater negative health impact [38]. This study considers pain arising in any part of the body, future research could examine whether the specific location of pain, or pain phenotype (e.g. widespread, regional or none) influence the association with RSA. The co-occurrence of sleep problems was high, with early awakening and delayed sleep onset rarely occurring in isolation (3.7% and 12.3% of individuals reported isolated symptoms respectively). While early awakening was most likely to co-occur with waking several times (93.1% of those with early awakening), delayed sleep onset often co-occurred with each of the other insomnia symptoms (60.7%, 73.0% and 77.0% respectively) suggesting it may be a marker of more severe symptoms. This may explain why delayed sleep onset was found to be most strongly associated with subsequent restricted participation.

This study has implications for primary care clinicians. Firstly, results support the idea that all adults consulting with musculoskeletal pain should be asked about concurrent insomnia, and patients who present with sleep problems should be asked about pain. The study indicates the importance of offering interventions to people where pain or sleep problems are identified. Current management options are limited and tend to target pain and insomnia separately: General practitioners can prescribe analgesics which target pain or, drugs which influence sleep (although these should not be prescribed long-term, due to the risk of addiction) and there is evidence to support such approaches [39]. However, there are known adverse effects to some medications that may limit their usefulness [39]. Psychological therapies and exercise are also known to be beneficial but are not commonly available [40]. A multidisciplinary holistic approach to management is likely to be the way forward [41]; however access to pain clinics which offer such an approach may be limited.

Approaches that reduce pain and insomnia is necessary to offer acceptable treatments for the large number of adults in the general population who experience both symptoms. Primary care clinicians provide support to patients to manage their symptoms and act as gate-keepers to onward referral. They should systematically explore the range of symptoms when a patient presents a single problem, such as pain or sleep disturbance, including symptoms of anxiety and depression. In addition, the clinician should explore social circumstances of the patient, including range of social contacts. The clinician can then give specific advice about the constellation of symptoms and problems that the patient may have, and which are likely to be interacting with each other. Such advice should include education about the possible interaction between sleep disturbance and pain and facilitation of increasing social activity, and engagement with the third sector to promote patient and self-management. Patients receiving interventions that target mood and physical ability have potential to improve social participation which acts as a buffer against morbidities such as cardiovascular disease [42] and maintains healthy ageing [16, 43].

## Conclusions

This study highlights co-occurring pain and insomnia as a prevalent problem in the general population, and suggests that those reporting both conditions experience greater impact upon social activity participation than those with only one condition. The study findings suggest co-occurring pain and insomnia may be an important target for clinical trials, rather than considering treatments for isolated symptoms. RSA risk factors which are potentially modifiable and treatable, such as anxiety and depression, present important targets for interventions and should be considered by those commissioning services. Further studies are warranted to determine how best to promote social activity participation in older people with comorbid pain and insomnia.

## Abbreviations

95%CI: 95% confidence interval
AUROC: Area under the receiver operating characteristic curve
OR: Odds ratio
RSA: Restricted social activity
SD: Standard deviation
